# Mitochondrial dysfunction in macrophages promotes inflammation and suppresses repair after myocardial infarction

**DOI:** 10.1172/JCI159498

**Published:** 2023-02-15

**Authors:** Shanshan Cai, Mingyue Zhao, Bo Zhou, Akira Yoshii, Darrian Bugg, Outi Villet, Anita Sahu, Gregory S. Olson, Jennifer Davis, Rong Tian

**Affiliations:** 1 Center for Mitochondria and Metabolism Center, Department of Anesthesiology and Pain Medicine and; 2 Department of Laboratory Medicine and Pathology, University of Washington School of Medicine, Seattle, Washington, USA.; 3 Center for Global Infectious Disease Research, Seattle Children’s Research Institute, Seattle, Washington, USA.; 4 Medical Scientist Training Program, University of Washington School of Medicine, Seattle, Washington, USA.

**Keywords:** Cardiology, Metabolism, Cardiovascular disease, Macrophages, Mitochondria

## Abstract

Innate immune cells play important roles in tissue injury and repair following acute myocardial infarction (MI). Although reprogramming of macrophage metabolism has been observed during inflammation and resolution phases, the mechanistic link to macrophage phenotype is not fully understood. In this study, we found that myeloid-specific deletion (mKO) of mitochondrial complex I protein, encoded by *Ndufs4*, reproduced the proinflammatory metabolic profile in macrophages and exaggerated the response to LPS. Moreover, mKO mice showed increased mortality, poor scar formation, and worsened cardiac function 30 days after MI. We observed a greater inflammatory response in mKO mice on day 1 followed by increased cell death of infiltrating macrophages and blunted transition to the reparative phase during post-MI days 3–7. Efferocytosis was impaired in mKO macrophages, leading to lower expression of antiinflammatory cytokines and tissue repair factors, which suppressed the proliferation and activation of myofibroblasts in the infarcted area. Mitochondria-targeted ROS scavenging rescued these impairments, improved myofibroblast function in vivo, and reduced post-MI mortality in mKO mice. Together these results reveal a critical role of mitochondria in inflammation resolution and tissue repair via modulation of efferocytosis and crosstalk with fibroblasts. These findings have potential significance for post-MI recovery as well as for other inflammatory conditions.

## Graphical abstract

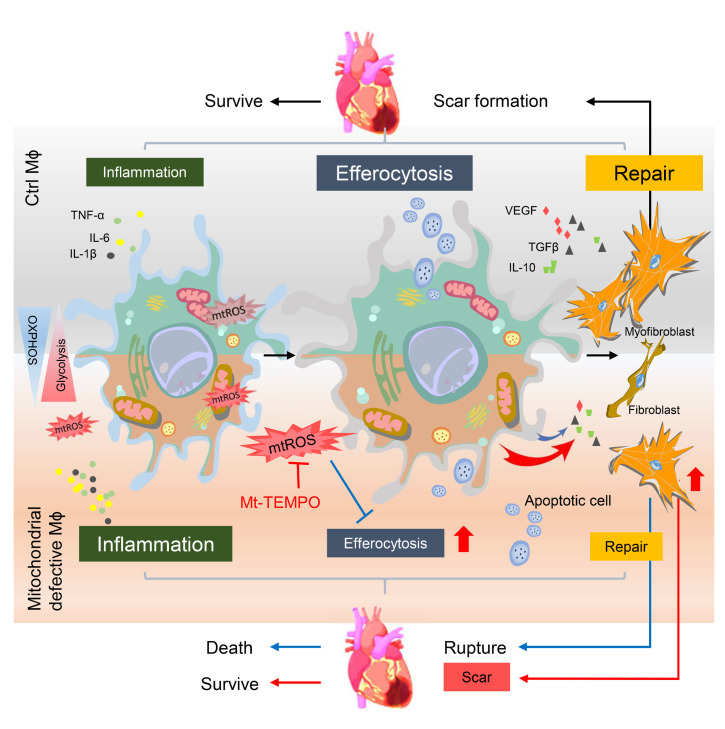

## Introduction

Innate immune cells, such as monocytes and macrophages, play vital roles in tissue injury and repair. Cell death caused by myocardial ischemia in the infarct region recruits and activates immune cells, which mount an inflammatory response in the early phase followed by tissue repair and scar formation (1). After acute myocardial infarction (MI), a majority of resident macrophages in the infarct zone die, and the area is populated by infiltrating neutrophils and macrophages derived from circulating monocytes (2, 3). Infiltration and activation of these cells lead to a strong inflammatory response, cytokine release, and further cell death immediately after MI (4). As the tissue repair commences, macrophage populations in the infarct zone evolve from a proinflammatory state to a prohealing one, which promotes collagen deposition, extracellular matrix remodeling, and scar formation (5, 6). While the critical role of monocytes and macrophages in the ischemic heart is well recognized (7), their complex and dynamic phenotype following MI makes therapeutic targeting extremely challenging (8, 9). A better understanding of mechanisms driving phenotypic changes of macrophages under pathological conditions will likely stimulate novel strategies for intervention.

It has been shown that macrophage phenotypes are linked to distinct metabolic profiles. Proinflammatory macrophages are predominantly glycolytic, whereas reparative macrophages are highly oxidative (10–12). Transcriptome analyses of macrophages in the myocardial infarct zone have shown a time-dependent reprogramming of mitochondrial and metabolic functions, which parallels the changes of macrophage function during post-MI remodeling of the heart (1). Mechanisms linking metabolism and macrophage function are, however, just now being explored. In the present study, we sought to unravel the mechanistic connection between metabolism and phenotype by investigating the wound-healing response in a mouse model of MI in the setting of defective mitochondrial complex I function in myeloid cells. We found that complex I deficiency promoted glycolysis and increased mitochondrial ROS (mtROS) in macrophages but did not cause inflammation in the absence of an additional stimulus. However, the metabolic shift primed myeloid cells for a larger inflammatory response, impaired efferocytosis, and decreased the transition to repair after MI. Furthermore, normalization of mtROS in the hearts of mKO mice reduced infarct rupture and improved survival after MI.

## Results

### Deletion of mitochondrial complex I protein Ndufs4 mimics the metabolic profile of inflammatory macrophages and exaggerated responses to LPS.

**Figure 1:**
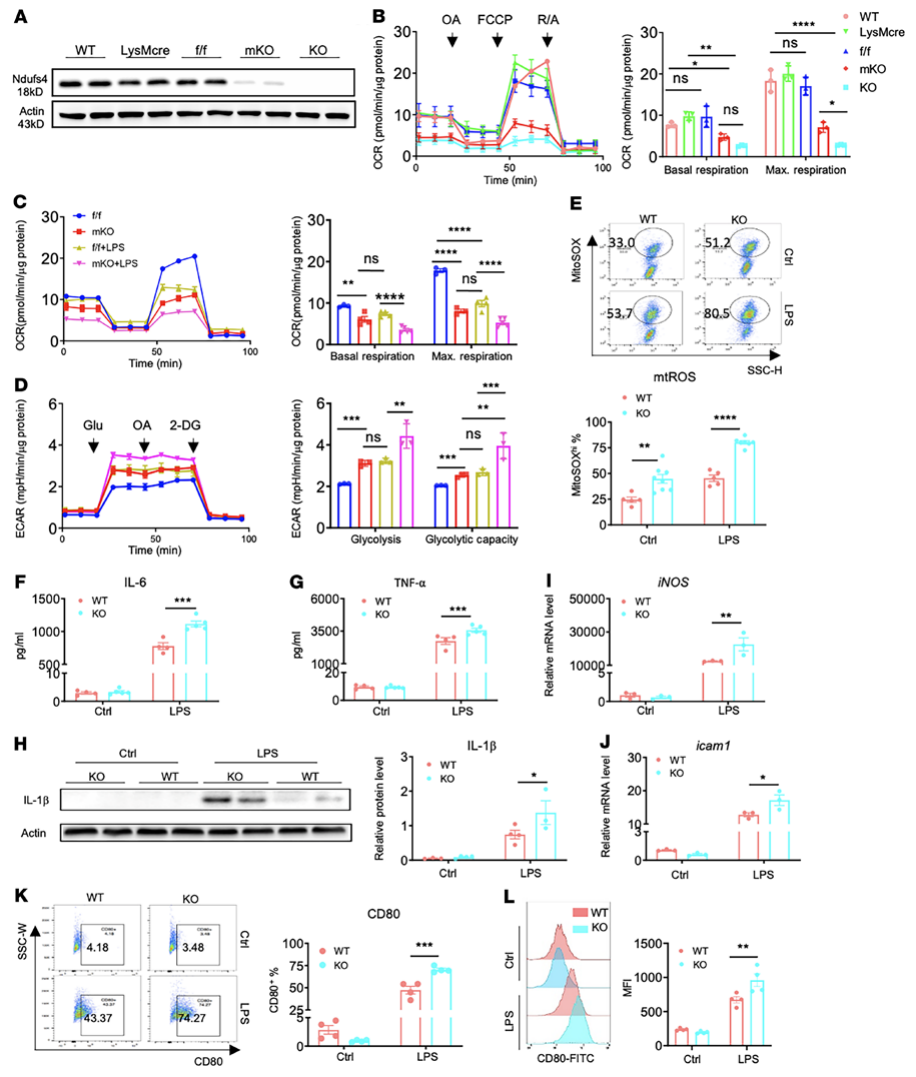
Deletion of the mitochondrial complex I protein Ndufs4 mimics the metabolic profile of inflammatory macrophages and exacerbates the response to LPS. (**A**) Ndufs4 protein expression levels in BMDMs from WT, LysMcre, f/f, mKO, and KO mice were detected by Western blotting. (**B**) Representative tracings of the OCR of BMDMs from the indicated groups of mice (left). The group average of basal and maximal (Max.) OCRs are shown (right). Vertical lines indicate time of addition of mitochondrial inhibitors oligomycin A (OA) (5 μM), FCCP (3 μM), or rotenone/antimycin A (R/A) (1 μM/1 μM). Experiments were repeated in 3 mice per group. (**C** and **D**) Mitochondrial respiration and glycolysis were measured according to the OCR (**C**) and the ECAR (**D**) in BMDMs treated with LPS (10 ng/mL) or vehicle for 6 hours. OCR and ECAR tracings are shown on the left. Average values at basal state and during maximum respiration or glycolysis (glycolytic capacity) are presented on the right. Experiments were repeated in 3–5 mice per group. (**E**) mtROS levels were measured by flow cytometry. MitoSOX was used as the ROS-sensitive dye in WT and KO macrophages pretreated with LPS (10 ng/mL) or vehicle for 6 hours. Representative flow cytometric analysis of MitoSOX fluorescence (upper) and the average of MitoSOX^hi^ percentage (lower) are shown. Experiments were repeated in 5–8 mice per group. SSC-H, side scatter height. (**F**–**H**) Protein levels in BMDMs treated with vehicle (PBS, control [Ctrl]) and LPS 100 ng/mL for 6 hours. IL-6 (**F**), TNF-α (**G**) levels were detected by ELISA, and IL-1β levels (**H**) were detected by Western blotting. Experiments were repeated in 4–5 mice per group. (**I** and **J**) Relative mRNA level of *iNOS* (**I**) and *Icam1* (**J**) in WT and KO BMDMs treated with 100 ng/mL LPS or PBS for 6 hours (*n* = 3/group). (**K**) Representative plots and quantification of CD80^+^ macrophages (percentage) in BMDMs treated with 100 ng/mL LPS or PBS for 24 hours. (**L**) Representative flow cytometry histogram and average (MFI) of CD80 staining. Experiments were repeated in 4 mice per group. All Data are presented as the mean ± SEM. *P < 0.05, **P < 0.01, ***P < 0.001, and ****P < 0.0001, by 1- or 2-way ANOVA.

We generated a mouse model with myeloid-specific deletion of *Ndufs4*, which encodes mitochondrial complex I protein (henceforth referred to as mKO mice), by crossing *LysMcre* and *Ndufs4^fl/fl^* mice (13, 14). In mKO mice, Ndufs4 protein levels in bone marrow–derived macrophages (BMDMs) or peritoneal macrophages (PMs) were reduced to a slight residual compared with Ndufs4-null (KO) samples (Figure 1A and Supplemental Figure 1A; supplemental material available online with this article; https://doi.org/10.1172/JCI159498DS1). Macrophages in *Ndufs4^fl/fl^* (henceforth referred to as f/f) or *LysMcre Ndufs4^+/+^* (henceforth referred to as LysMcre) mice had normal Ndufs4 protein levels, as in WT C57BL/6J (henceforth referred to as WT) mice. Both f/f and LysMcre mice were used as controls in this study. Mitochondrial respiration (oxygen consumption rate; [OCR]) of mKO macrophages was effectively reduced in both the basal state and during stimulation by carbonyl cyanide-*p*-trifluoromethoxyphenyl-hydrazon (FCCP) (Figure 1, B and C, and Supplemental Figure 1B), whereas mitochondrial mass in BMDMs was unaffected by Ndufs4 deletion (Supplemental Figure 1C). When subjected to LPS, control macrophages switched from oxidative metabolism to glycolysis, as demonstrated by a decrease in the OCR accompanied by an increase in the extracellular acidification rate (ECAR) (Figure 1, C and D). Interestingly, the metabolic profile of mKO macrophages in the basal state mimicked that of the LPS-treated controls. LPS further reduced the OCR and elevated the ECAR in mKO macrophages (Figure 1, C and D). We also observed a greater upregulation of glycolytic gene expression in mKO macrophages after LPS treatment (Supplemental Figure 1, E–K). Similarly, mtROS levels were increased in mKO macrophages or by LPS treatment, and the highest level of mtROS was observed in LPS-treated KO macrophages (Figure 1E). Mitochondrial membrane potential was moderately reduced in KO but not affected by LPS treatment (Supplemental Figure 1D). Taken together, mKO recapitulated the metabolic switch in proinflammatory macrophages and enhanced the metabolic response to LPS.

Despite the baseline shift toward glycolysis, mKO BMDMs showed no increases in the proinflammatory markers in the basal state, suggesting that metabolic changes alone did not cause inflammation (Figure 1, F–L, and Supplemental Figure 1, M–S). However, upon LPS treatment, KO BMDMs produced more proinflammatory cytokines, i.e., IL-6, TNF-α, and IL-1β (Figure 1, F–H) and had higher mRNA levels of *iNOS* and *icam1* (Figure 1, I and J). Moreover, LPS challenge resulted in higher expression of CD14 in mKO macrophages (Supplemental Figure 1N) and a higher percentage of CD80^+^ macrophages in KO compared with control cells (Figure 1K). Together, these results demonstrated that the metabolic switch to glycolysis in macrophages was insufficient to initiate inflammation but modulated inflammatory responses upon LPS stimulation.

### Myeloid-specific mitochondrial deficiency worsens post-MI outcomes.

**Figure 2:**
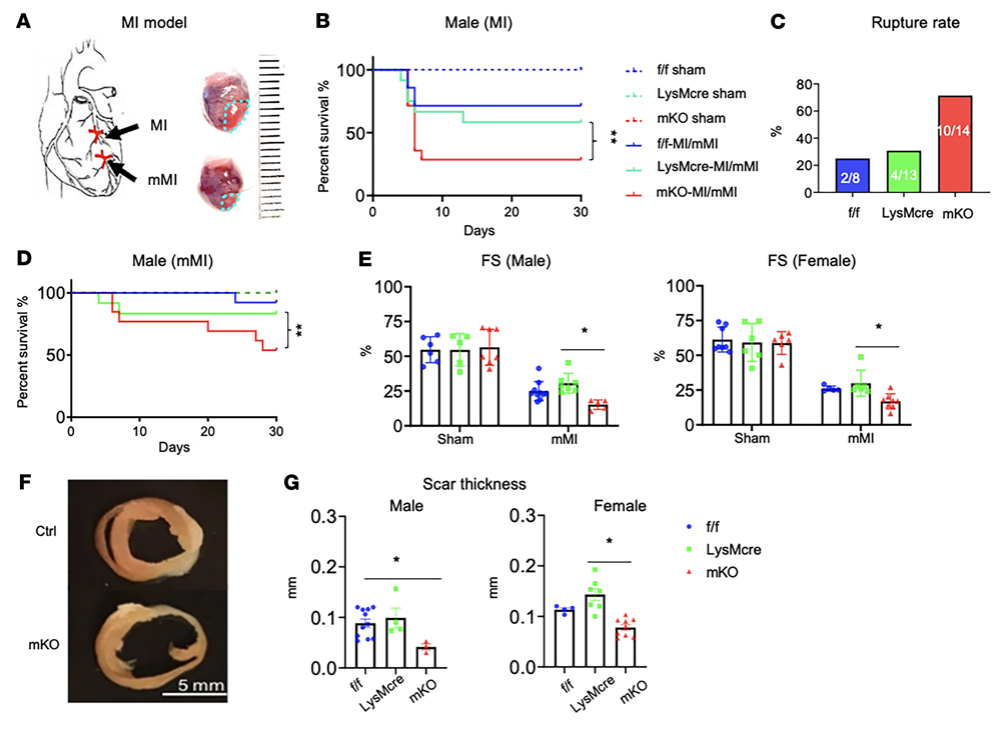
Myeloid-specific mitochondrial deficiency worsens post-MI outcomes. (**A**) Pictorial description of the MI model by ligation of the left coronary artery. (**B**) Survival rate in the 30 days following MI or sham operation for the indicated groups. (**C**) Rate of cardiac rupture out to 7 days after MI. (**D**) Survival rate of male mice following ligation at a lower site along the left descending coronary artery, as shown in **A**, to induce a smaller ischemic area (mMI). Survival rates were compared by Gehan–Breslow–Wilcoxon test with a *P* value of less than 0.01 considered statistically significant. (**E**) LV FS after mMI assessed by echocardiography at day 30 in male and female mice (*n* = 4–9/group). (**F**)Mid-ventricular sections from a f/f and a mKO perfusion-fixed heart at post-MI day 30. (**G**) Male and female scar thickness measured in sections with Masson’s trichrome stain at day 30 after mMI (*n* = 4–11/group). Data are presented as the mean ± SEM. *P < 0.05 and **P < 0.01, by 1-or 2-way ANOVA.

To determine the in vivo consequence of myeloid-specific mitochondrial impairment, we subjected mKO mice to MI (Figure 2A). It is known that myeloid cells play important roles in both early inflammatory and late healing phases after a MI (15). Given the exaggerated response to LPS, we hypothesized that mKO would mount a more severe inflammatory response in the infarcted region. In the first week after MI, we observed a mortality rate of 71% for mKO mice versus 30% for f/f and LysMcre controls (Figure 2, A and B, and Supplemental Table 1). Necropsy confirmed an infarct rupture in the majority of the deaths (Figure 2C). To study tissue repair and healing after MI, we generated a cohort of mice with a moderate infarct size (moderate MI [mMI]) to increase the yield of mice surviving beyond the first week (Supplemental Table 1). In the mMI cohort, all female mice survived out to 30 days, although the mortality rate for male mKO mice remained higher than that of male control mice (Figure 2D). The sex difference in survival rates was consistent with the literature reporting that female mice are protected from post-MI cardiac rupture compared with male mice (16). Both hormone levels and cellular intrinsic properties could account for the sex differences in stress responses (16, 17). As females of both genotypes were protected from cardiac rupture, the mechanisms would likely be independent of mitochondrial function in myeloid cells. Echocardiography performed 30 days after MI detected a greater impairment of left ventricular (LV) function in both male and female mKO mice (Figure 2E and Supplemental Figure 2, C–F). In infarcted mice that survived to 30 days, the scars were significantly thinner in mKO mouse hearts, and the infarct size tended to be larger (Figure 2, F and G, and Supplemental Figure 2, G and H). Collectively, the results suggested that mKO promoted cardiac injury and impaired scar formation after MI.

### Heightened inflammation is coupled with increased macrophage cell death and failed transition to a reparative phenotype in mKO mice after MI.

**Figure 3:**
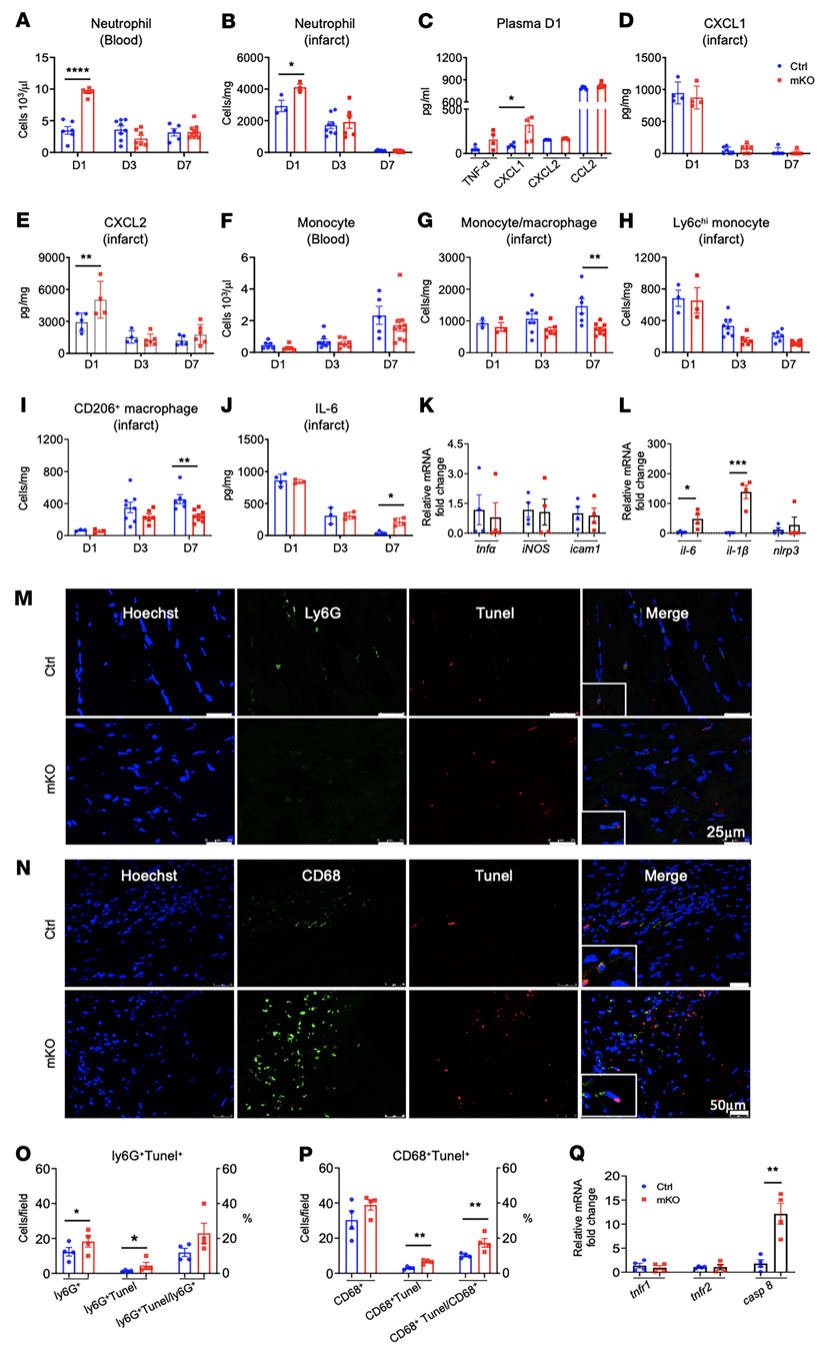
Heightened inflammation is coupled with increased macrophage death and failed transition to a reparative phenotype in mKO mice after MI. (**A** and **B**) Flow cytometric quantification of neutrophils in blood (**A**) and infarcted tissue (**B**) at days 1, 3, and 7 (D1, D3, D7) after mMI (*n* = 3–8/group). (**C**–**E**) Cytokine/chemokine levels of TNF-α, CXCL1, CXCL2, and CCL2 in plasma on day 1 after mMI (**C**), as well as levels of CXCL1 (**D**) and CXCL2 (**E**) chemokines in infarcted tissue at post-MI days 1, 3, and 7 were detected by ELISA (*n* = 4–6/group). (**F**–**I**) Flow cytometric quantification of monocytes (CD45^+^CD11b^+^ly6G^–^) in blood (**F**) and monocytes and macrophages in infarcted tissue (**G**) at post-mMI days 1, 3, and 7. Ly6C^hi^ monocytes (**H**) and CD206^+^ macrophages (**I**) in infarcted tissue at post-MI days 1, 3, and 7 (*n* = 3–10/group). (**J**) IL-6 cytokine levels in infarcted tissue at post-MI days 1, 3, and 7 were detected by ELISA (*n* = 3–4/group). (**K**–**L**) mRNA levels of selected genes involved in inflammatory responses in cM cells isolated from infarcted tissue at day 3 after MI. RNA was extracted from cM cells and then subjected to quantitative PCR (qPCR) (*n* = 4/group). Data are presented as the mean ± SEM. (**M**–**Q**) Detection of apoptotic neutrophils and macrophages in mouse heart at post-MI day 3. (**M**) Frozen sections of heart tissue from LysMcre and mKO mice were double stained for Ly6G (green) and Tunel (red), and nuclei were stained with Hoechst (blue) Scale bars: 25 μm. (**N**) Same sections as in **M**, stained for CD68 (green) and Tunel (red); nuclei were stained with Hoechst (blue) Scale bars: 50 μm. (**O**) Quantitative analysis of ly6G^+^ (green) and ly6G^+^Tunel^+^ cells per field (left *y* axis) and percentage of ly6G^+^Tunel^+^ cells per Ly6G^+^ nuclei in the infarcted area (right *y* axis). (**P**) Quantitative analysis of CD68^+^, CD68^+^Tunel^+^ cells per field (left *y* axis) and percentage of CD68^+^Tunel^+^ per CD68^+^ nuclei in the infarcted area (right *y* axis) (*n* = 4/group). Data are presented as the mean ± SEM. (**Q**) mRNA levels of selected genes involved in apoptotic cell death in cM cells isolated from infarct tissue at post-MI day 3 (*n* = 4/group). *P < 0.05, **P < 0.01, and ***P < 0.001, by 2-tailed Student’s *t* test.

Under baseline conditions, mKO mice demonstrated no signs of inflammation, and the number of circulating myeloid cells was similar in f/f, LysMcre, and mKO mice (Supplemental Figure 3, A–C). On day 1 after MI, we observed higher neutrophil levels in the circulation and in the infarcted area in mKO mice (Figure 3, A and B). Proinflammatory chemokines and cytokines in the plasma (CXCL1) and in the infarcted area (CXCL2) were also increased in mKO mice on day 1 after MI (Figure 3, C–E).

Despite evidence of increased inflammatory responses and the expectation that there might be more monocyte/macrophage recruitment, the numbers of monocytes or macrophages in the circulation or the infarcted region of mKO mice were similar to those of controls on day 1 and day 3 after MI (Figure 3, F–I). There were actually fewer monocytes/macrophages in the infarcted region in mKO mice on day 7 following MI (Figure 3G). Furthermore, we observed significantly fewer CD206^+^ macrophages in mKO infarcts (Figure 3I), accompanied by higher levels of the proinflammatory cytokine IL-6 on day 7, suggesting that the transition to inflammation resolution was delayed in the mKO hearts (Figure 3J). In CD11b^+^ myeloid cells isolated from the infarcted region of mKO hearts on post-MI day 3, gene expression of *il-6* and *il-1β* was significantly higher (Figure 3, K and L). Immunofluorescence staining revealed a significantly higher number of neutrophils and macrophages undergoing apoptosis on day 3 after MI in mKO hearts (Figure 3, M–P). Although the expression of TNF-α receptors (TNFR1/2) in cardiac myeloid (cM) cells were not different between mKO and control mice on day 3 after MI, caspase 8 expression was elevated in the mKO group, suggesting that a mitochondrial defect promoted cell death via signaling transduction downstream of TNFR1 (Figure 3Q). These results suggest that myeloid-specific mitochondrial complex I deficiency not only promoted an inflammatory response and cell death in the infarcted region at the early post-MI stage, but also impaired the transition of infiltrating macrophages to reparative phenotypes during the healing phase.

### Defective efferocytosis blunts antiinflammatory responses in mKO macrophages.

**Figure 4:**
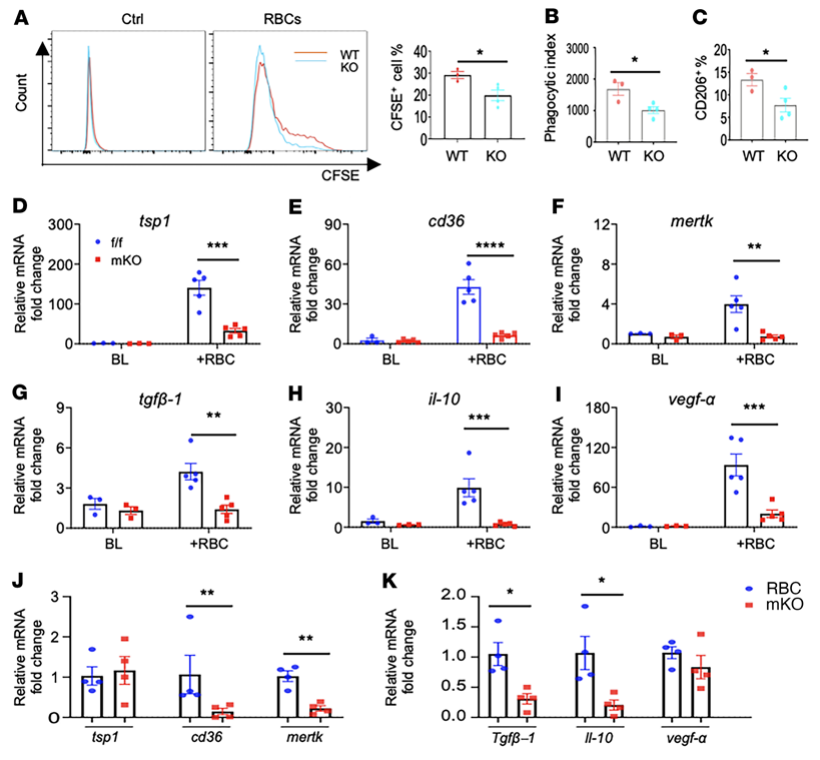
Defective efferocytosis blunts antiinflammatory responses in mKO macrophages. (**A**–**C**) Flow cytometric analysis of efferocytosis in BMDMs. BMDMs from WT and mKO mice were treated with CFDA-SE–prelabeled apoptotic RBCs for 3 hours. Representative histograms of total CFDA-SE^+^ counts (left) and quantitation of phagocytic macrophages (right) (**A**). Quantitation of the phagocytic index (**B**) and the percentage of CD206^+^ phagocytic macrophages (**C**). All experiments were repeated in 3–4 mice per group. Data are presented as the mean ± SEM. *P < 0.05, by Student’s *t* test. (**D**–**I**) qPCR analysis of mRNA levels in macrophages upon coculturing with apoptotic RBCs for 8 hours. All experiments were repeated using macrophages from 3–5 mice per group. Data are presented as the mean ± SEM. **P < 0.01, ***P < 0.001, and ****P < 0.0001, by 2-way ANOVA. (**J** and **K**) mRNA levels of selected genes involved in efferocytosis in cM cells isolated from infarcted tissue at day 3 after MI (*n* = 4/group). Data are presented as the mean ± SEM. *P < 0.05 and **P < 0.01, by 2-tailed Student’s *t* test.

Clearance of dead cells by professional phagocytes, a process known as efferocytosis, is a critical step in the resolution of inflammation and initiation of tissue repair. Since we observed increased cell death and a delayed transition to the reparative phase in mKO mice, we hypothesized that defective efferocytosis might account for the increased post-MI mortality rate of mKO mice. To assess the efferocytotic function of mKO macrophages, we subjected BMDMs to CFSE-prelabeled apoptotic RBCs for 3 hours and measured the percentage of CFSE^+^ cells (phagocytic efficiency) and CFSE content in the positive cells (phagocytic index). We found that both measurements were reduced in mKO macrophages (Figure 4, A and B). Since efferocytosis is known to induce phenotypic changes in macrophages toward a reparative phenotype, we next investigated whether mKO macrophages are impaired in switching toward a reparative phenotype after efferocytosis. We observed that mKO BMDMs subjected to apoptotic RBCs had a lower percentage of CD206^+^ reparative cells (Figure 4C). This finding was consistent with reduced CD206^+^ macrophages in the infarcted region of mKO hearts (Figure 3I), supporting the notion that impaired efferocytosis contributed to suppression of the reparative phenotype in mKO macrophages. Efferocytosis stimulated the expression of molecules for cell adhesion, e.g., *cd36*, thrombospondin 1 (*tsp1*), and MER proto-oncogene tyrosine kinase (*mertk*), or antiinflammatory mediators, e.g., TGF-β1 (*tgfβ1*), *il-10*, and VEGF-α (*vegf-α*), in control macrophages to a significantly greater degree than in mKO macrophages (Figure 4, D–I). In support of the in vitro results, the expression of *cd36*, *mertk*, *il-10*, and *tgfβ*1 genes was also lower in CD11b^+^ myeloid cells isolated from the infarcted region of mKO hearts compared with controls on post-MI day 3 (Figure 4, J–K). The causative role of efferocytosis in this process was confirmed by inhibiting efferocytosis with increasing doses of cytochalasin B and observing a dose-dependent decrease in the gene expression of these proteins in control macrophages (Supplemental Figure 4). Given the important role of these proteins in wound healing, our results suggested that defective efferocytosis in mKO macrophages could lead to impaired repair of the infarcted region in mKO hearts.

### Activation and proliferation of cardiac myofibroblasts are suppressed in mKO mice after MI.

**Figure 5:**
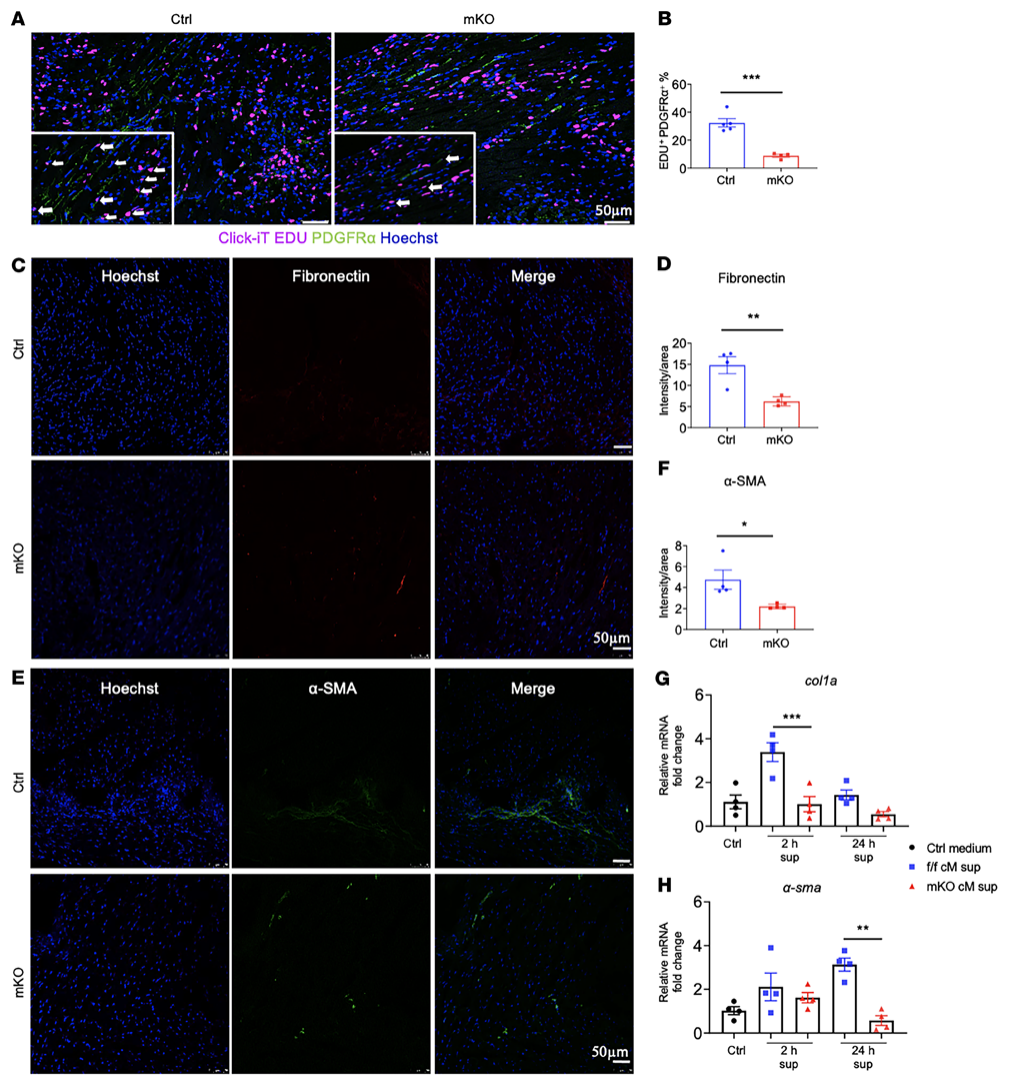
Activation and proliferation of cardiac myofibroblasts is suppressed in mKO mice after MI. (**A**) Representative immunofluorescence images of CF proliferation. LysMcre and mKO mouse hearts were prelabeled with 100 mg/kg EdU 24 hours and 9 hours before harvesting on post-MI day 3. Cryosections of the heart were assessed by Click iT kit to detect Edu (pink) and PDGFRα (green). Nuclei are stained with Hoechst (blue). Scale bars: 50 μm. Arrows show proliferating myofibroblasts, i.e., PDGFRα^+^ and EdU^+^ cells. (**B**) Quantification of proliferating myofibroblasts as a percentage of all PDGFRα^+^ cells in the infarcted region (*n* = 4–5/group). Dots represent biological replicates, and data represent the mean ± SEM. ***P < 0.001, by unpaired, 2-tailed Student’s *t* test. (**C**) Immunofluorescence imaged of fibronectin in infarcted heart tissue at post-MI day 7. Scale bars: 50 μm. (**D**) Quantitative morphometry of immunostaining, in which the relative abundance of the stained area was calculated by averaging the results from multiple independent images from f/f and mKO mice (*n* = 4/group). **P < 0.01, by unpaired, 2-tailed Student’s *t* test. (**E**) Representative immunofluorescence images of α-SMA staining in the infarcted region at post-MI day 7: fibronectin (red), α-SMA (green), Hoechst (blue). Scale bars: 50 μm. (**F**) Quantification of α-SMA^+^ staining in heart tissue at post-MI day 7 (*n* = 4 per group). *P < 0.05, by unpaired, 2-tailed Student’s *t* test. (**G** and **H**) mRNA expression levels of *col1α* (**G**) and *α-SMA* (**H**) were determined by qPCR in CFs treated with supernatants of spent medium of cM cells or control medium. cM cells were isolated from infarcted heart tissue at post-MI day 3 and then cultured in DMEM with 10% FBS for 2 hours or 24 hours. The supernatants of the spent medium or control medium were collected and added to the CF culture for an additional 24 hours. RNA was extracted from CFs and then subjected to qPCR (*n* = 4/group). Dots represent biological replicates, and data represent the mean ± SEM. **P < 0.01 and ***P < 0.001, by 1-way ANOVA.

The production of antiinflammatory and proreparative factors, such as TGF-β, is an important mechanism for the crosstalk between macrophages and fibroblasts during wound healing (18, 19). To determine the in vivo relevance of the impaired antiinflammatory response observed in mKO BMDMs, we compared the activation and proliferation of cardiac fibroblasts (CFs) in the infarcted area of control and mKO mice 3 days after MI. Mice were injected with 5-ethynyl-2′-deoxyuridine (EdU) (100 mg/kg, i.p.) 24 hours and 9 hours before tissue harvesting to label proliferating cells. Cells with colocalization of EdU and PDGFRα, a cell-surface marker for resident CFs (20), were quantified in the infarcted area to assess fibroblast proliferation. In myocardial sections, we found a significantly lower percentage of PDGFRα^+^ and EdU^+^ cells in the infarcted area of mKO hearts 3 days after MI (Figure 5, A and B). Consistent with reduced proliferation, expression of fibronectin and α–smooth muscle action (α-SMA), markers of fibroblast activation, was also significantly lower in the infarcted area of mKO hearts (Figure 5, C–F). To assess the crosstalk between macrophages and fibroblasts, we treated CFs with secretome of CD11b^+^ cells isolated from MI hearts. Secretome from the control-MI group elicited a robust increase in the expression of *col1a* and *α-sma* in CFs, suggesting that these fibroblasts were activated. This response was absent when mKO secretome was used (Figure 5, G and H). These results collectively suggested that the lack of stimulation from reparative macrophages impaired the proliferation and activation of CFs in post-MI mKO hearts.

### Mitochondria-targeted ROS scavenging improves macrophage function and partially rescues mortality of mKO mice after MI.

**Figure 6:**
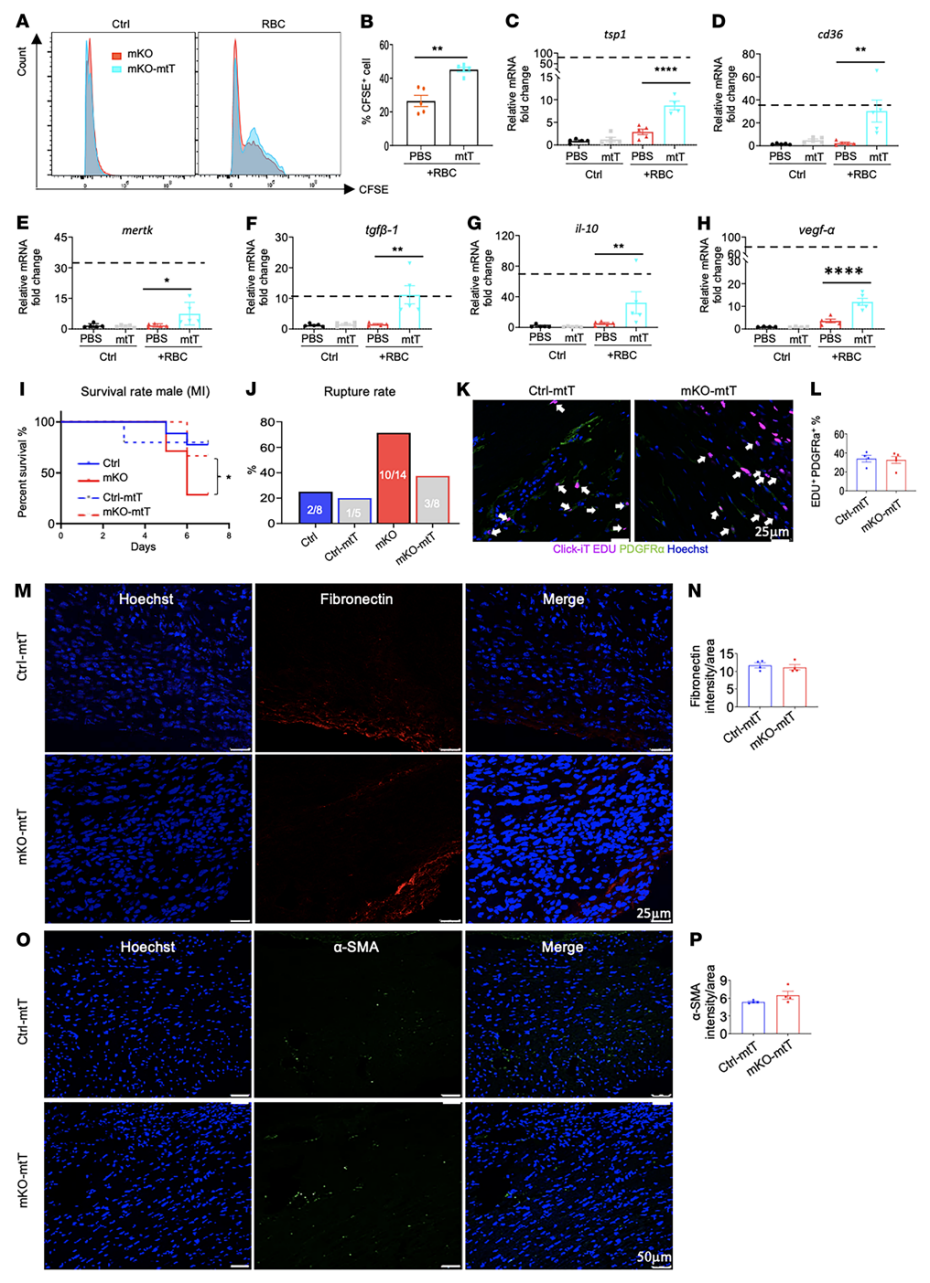
Mitochondria-targeted ROS scavenging improves macrophage function and reduces mortality in mKO mice after MI. (**A** and **B**) Apoptotic RBCs were labeled with CFDA-SE and cocultured with mKO BMDMs (±1 μM mtT) for 3 hours. Representative histograms of total CFDA-SE^+^ cell counts (**A**) and quantitation of phagocytic macrophages (**B**) (*n* = 5/group). **P < 0.01, by 2-tailed Student’s *t* test. (**C**–**H**) mRNA levels in mKO BMDMs after mtT (1 μM) or vehicle treatment and coculturing with or without apoptotic RBCs for 8 hours. All values are presented as the fold change relative to untreated control BMDMs. Dashed lines indicate mRNA levels in the control BMDMs cocultured with apoptotic RBCs. Data are shown as the mean ± SEM. All experiments were repeated in BMDMs from 4–5 mice per group. *P < 0.05, **P < 0.01, and ****P < 0.0001, by 2-way ANOVA. (**I**) Survival rates of mKO mice with or without administration of mito-TEMPO (10 mg/kg day) out to post-MI day 7. *P < 0.05, by Gehan–Breslow–Wilcoxon test. (**J**) Cardiac rupture rate during the first 7 days after MI. (**K**) Representative immunofluorescence images of cardiac myofibroblast proliferation at post-MI day 3. Proliferating myofibroblasts were identified (white arrows) as PDGFRα^+^ (green) and EdU^+^ (pink). Nuclei are stained with Hoechst (blue). Scale bar: 25 μm. (**L**) Quantitation of PDGFRα^+^ and EdU^+^ cells as a percentage of total PDGFRα^+^ cells in the infarcted region (*n* = 4–5/group). Dots represent biological replicates, and data represent the mean ± SEM. (**M**) Immunofluorescence images of fibronectin in infarcted heart tissue at post-MI day 7. Scale bars: 25 μm. (**N**) Quantitative morphometry of immunostaining, in which the relative abundance of the stained area was calculated by averaging the results from multiple independent images of heart sections (*n* = 4/group). (**O**) Representative immunofluorescence images of α-SMA in the infarcted heart section at post-MI day 7: fibronectin (red), α-SMA (green), and Hoechst (blue). Scale bars: 50 μm. (**P**) Quantification of α-SMA^+^ levels in heart tissue at post-MI day 7 (*n* = 4/group). Dots represent biological replicates, and data represent the mean ± SEM.

As mKO macrophages produced more mtROS (Figure 1E), we next determined whether scavenging ROS in the mitochondria could improve efferocytosis. After treatment with the mitochondria-targeted antioxidant mito-TEMPO (mtT), mKO BMDMs showed increased phagocytic activity upon exposure to apoptotic RBCs (Figure 6, A and B). Moreover, restoration of efferocytosis in mKO was accompanied by upregulation of mRNA expression levels of the adhesion molecules *tsp1, cd36,* and *mertk*, the growth factors *tgfβ1* and *vegf-α*, as well as the antiinflammatory cytokine *il-10* (Figure 6, C–H). Treatment with mtT slightly reduced glycolysis but did not correct the mitochondrial respiration in mKO macrophages (Supplemental Figure 5, A–D). Collectively, these results suggested that increased mtROS could be a potential link between defective mitochondrial function and impaired efferocytosis in mKO macrophages. To test whether normalization of efferocytosis could improve post-MI outcomes of mKO mice in vivo, we administrated mtT (10 mg/kg, i.p.) to mice immediately after MI surgery. Over the 7 days after MI, the survival of mKO mice treated with mtT increased from 29% to 62.5% (Figure 6I). The incidence of cardiac rupture in mKO mice was reduced by approximately 2-fold with mtT treatment (Figure 6J). Moreover, mtT improved myofibroblast proliferation (Figure 6, K and L) and increased fibronectin and α-SMA levels in the infarcted region (Figure 6, M–P). These results corroborated the in vitro study and suggested a critical role of mitochondrial function in the communication between macrophages and fibroblasts during post-MI cardiac repair.

## Discussion

In this study, we demonstrated that a metabolic shift from oxidative metabolism to glycolysis primed myeloid cells for a greater inflammatory response, although it did not induce inflammation at baseline. In mice with a myeloid cell–restricted mitochondrial deficiency (mKO), MI triggered a greater inflammatory response and impaired macrophage transition to a reparative phenotype, leading to poor wound healing and high mortality. Mechanistically, we found that excessive mtROS impaired macrophage efferocytosis and reduced antiinflammatory and prohealing signals. Mitochondria-targeted ROS scavenging improved efferocytosis, restored the crosstalk between macrophages and CFs, and reduced infarct rupture in mKO mice. Our study, thus, revealed a critical role of mitochondrial function in myeloid cells during the transition from inflammation to resolution in a mouse model of MI.

One characteristic of inflammatory macrophages is the shift of oxidative metabolism to aerobic glycolysis for energy production (21). An increased flux of glycolytic metabolites into the pentose phosphate pathway provides NADPH to fuel ROS production by NADPH oxidase, thereby promoting antimicrobial function (12, 22). Oxidative metabolism in mitochondria under this condition is downregulated and diverted from generating ATP to producing ROS (23). By inducing mitochondrial complex I deficiency, we were able to recapitulate the metabolic shift in macrophages in this study. However, we found that such a shift, per se, did not initiate inflammation but rather primed the cells for a greater inflammatory response during LPS stimulation or after MI, suggesting an important modulatory, but not triggering, role of cell metabolism in innate immunity. The priming mechanism remains unclear. Although the expression of TLRs or TNF-α receptors is unaltered in mKO, increased expression of CD14 in mKO mice could account for an exacerbated response to LPS stimulation. On the other hand, our study showed that metabolic flexibility was required for inflammation resolution, during which restoration of mitochondrial function promoted antiinflammatory signaling. A previous study has shown robust expression of mitochondrial genes in cardiac macrophages during the transition to wound healing after MI (1). In the present study, the inability to recover mitochondrial function in cardiac macrophages led to increased cell death and failure to develop a reparative phenotype. Collectively, these observations demonstrate an essential role of dynamic changes in immune cell metabolism and identify metabolic flexibility as a likely target for future interventions.

A main finding of the study is that defective mitochondrial function impaired macrophage efferocytosis and reduced the production of tissue repair factors. Although mechanistically similar to phagocytosis, efferocytosis avoids antigen presentation and inflammation through efficient degradation of internalized apoptotic cells (24). Upon apoptotic cell uptake, macrophages are reprogrammed to a “resolving” phenotype that prevents secondary necrosis, limits the production of inflammatory cytokines, and increases the release of reparative mediators. Therefore, effective efferocytosis is not only crucial in the maintenance of tissue homeostasis but also in regulating and inducing a tissue repair response. A study by Zhang et al. suggested that the capacity of mitochondria to process the efferocytotic cargo was crucial for antiinflammatory function of macrophages in the infarcted heart (25). Corroborating and further expanding the concept, the present study shows that normal mitochondrial function was required for efferocytosis. The Zhang study found that an inability to oxidize fatty acid cargo following efferocytosis caused hyperacetylation and inhibition of PBX1, a transcription factor promoting IL-10 expression (25). Our study, on the other hand, demonstrates that excessive mtROS impaired efferocytosis function. In addition, an earlier study by Wang et al. found that mitochondrial calcium sequestration due to defective fission could also impair efferocytosis and exacerbate atherosclerosis in mice (26). These observations collectively demonstrate that mitochondrial function is a key determinant of efferocytosis and downstream cascades, likely via multiple mechanisms depending on the pathological conditions.

Cardiac wound healing is characterized by different steps involving the generation of new vessels, fibroblast proliferation and differentiation, the deposition of extracellular matrix (ECM), and reepithelialization. Macrophages have been shown to play a pivotal role in orchestrating tissue repair including activation of fibroblasts (27, 28). Such an ability is significantly compromised in mitochondria-deficient macrophages, as shown here, partly due to defective efferocytosis and, consequently, reduced expression of prohealing cytokines and tissue growth factors, such as TGF-β. In this study, activation of myofibroblasts was improved when efferocytosis was restored by scavenging of excessive mtROS. The result does not rule out the possibility that mitochondria-targeted ROS scavenging in other cell types also contributed to the benefits (29, 30). However, mito-TEMPO treatment only slightly reduced the infarct rupture in control mice with intact macrophage mitochondrial function, suggesting that excessive mtROS in mKO macrophages is an important pathological mechanism. Moreover, mtROS scavenging has been shown to reduce chronic remodeling of the heart in animal models (31). Whether excessive mtROS in macrophages plays a role in the development of heart failure independent of wound healing warrants further investigation.

This study has a number of limitations, specifically, LysMcre-driven gene deletion affects both neutrophils and monocytes/macrophages. There was indeed a higher number of neutrophils on day 1 after MI in mKO mice, which could have contributed to the poorer post-MI outcomes (32–34). The cause of increased neutrophil recruitment in mKO hearts is not clear. We observed an increase of CXCL2 in the infarcted area of mKO hearts, suggesting that a heightened chemoattractant signal could be the culprit. Recent studies suggest that chemokine signaling from cardiac-resident CCR2^+^ macrophages after MI can mobilize CCR2^+^ inflammatory monocytes and extravasation of neutrophils (35, 36). These observations raise the possibility that mitochondrial deficiency in CCR2^+^ cardiac-resident macrophages promotes the recruitment and infiltration of neutrophils and monocytes after MI. Alternatively, mitochondrial dysfunction in neutrophils could alter their function, resulting in a greater mobilization in mKO mice after MI. Compared with other immune cells, neutrophils have lower oxidative metabolism (37), and the role of mitochondria in neutrophil function is poorly understood. These observations, however, advocate for future studies specifically focused on mitochondrial function in neutrophils.

In conclusion, we demonstrated what we believe to be a pivotal role of mitochondrial function in modulating the inflammatory response and resolution during recovery after an MI. Specifically, we found that the efferocytic pathway was a key downstream target through which mitochondrial function of macrophages regulated tissue injury and repair.

## Methods

### Generation of mice with myeloid cell–specific loss of Ndufs4.

We bred *LysMcre* mice (The Jackson Laboratory, stock no. 004781) with *Ndufs4*-floxed mice (13) to generate myeloid-specific deletion of *Ndufs4* (mKO) and the corresponding *Ndufs4^fl/fl^* and *LysMcre/Cre* littermate controls. Macrophages from Ndufs4-null mice were used as positive controls in some experiments as indicated.

### MI model in mice.

All mice (male and female; 8–12 weeks old; 20–28 g) used for this study were on a C57BL/6J background. Permanent left anterior descending artery (LAD) ligation or a sham operation was performed on experimental animals as described in a previously published protocol (38). Briefly, the mice were anesthetized by inhalation of 2% isoflurane. A small skin cut (1.2 cm) was made over the left chest to expose the heart, and a 7-0 silk suture was used for permanent ligation of the LAD. The suture was passed approximately 2 mm (for the MI model) or 3 mm (for the mMI model) below the base of the left auricle. Mice that did not survive the first 24 hours after the surgery were excluded from analysis. Sham-operated animals underwent the same procedure without coronary artery ligation. For immunostaining and histology analysis, hearts were excised and arrested in diastole with buffer containing a high concentration of potassium chloride before being fixed in formalin and prepared for paraffin or OCT embedment and sectioning. In the rescue experiment, 10 mg/kg Mito-TEMPO (MilliporeSigma, SML0737) was injected i.p. daily and started immediately after MI surgery. The experimentalists remained blinded to the genotypes until analysis was complete. Both male and female mice were used in all experiments, and mice were randomly assigned to the groups.

### Echocardiography.

Cardiac function was assessed by echocardiography performed on a Vevo 2100 Ultrasound system (VisualSonics) when mice were mildly anesthetized and maintained with 0.5% isoflurane at a heart rate of 500–600 beats/min. Short-axis M-mode was used for the measurement of systolic and diastolic ventricular diameters and wall thicknesses. LV contractile function was assessed as fractional shortening (FS).

### Infarct size and scar thickness determination.

Heart specimens were cleared of blood with ice-cold PBS and then fixed in 4% formalin, embedded in paraffin, and cut into 5 μm thick sections. Fixed hearts were sectioned along the short axis into 1 mm slices, and a 5 μm section cut from each slice was stained with Masson’s trichrome to determine infarct size. Infarct size was calculated as the average of the infarcted circumference divided by the LV circumference × 100 in all slices, as described previously (39, 40). The data were analyzed using ImageJ software (NIH).

### Isolation of immune cells from the blood and hearts.

Peripheral blood (50 μL) was taken immediately after cervical dislocation for flow cytometric analysis. RBCs were lysed with 2 mL ACK lysing buffer (Gibco, Thermo Fisher Scientific, A10492-01) for 10 minutes. Excised LV infarcted tissue was rinsed and immediately minced and digested by warm digestion buffer containing collagenase type 2 (0.05%, Worthington Biochemical, LS004177) and DNase 1 (60 U/mL DNase 1, MilliporeSigma, 10104159001), followed by shaking for 5 minutes in 37°C. A single-cell suspension was generated and filtered through a 40 μm filter into ice-cold stopping buffer containing 10% FBS in 5 mL PBS. This process was repeated, with the tissue remaining in the filter for a total of 5 digestions. Cells were centrifuged (400*g*, 5 min), and RBCs were lysed with 2 mL ACK lysing buffer (Gibco, Thermo Fisher Scientific, A10492-01) for 5 minutes. Cells were then washed twice with PBS. Immune cells isolated from blood and infarcted heart tissue were resuspended in 100 μL staining buffer (1% PBS in 2 mM EDTA). The cells were incubated with 1% CD16/CD32 for 10 minutes at room temperature followed by incubation of a 1% antibody mixture on ice for 20 minutes. Cells were then washed twice with iced PBS, counted, and fixed. For flow cytometric analysis, data were acquired with an Aurora flow cytometer (Cytek) and analyzed with FlowJo 10.7.1 software (FlowJo). Viability stain 7-AAD was used to identify live cells. The gating strategies are shown in Supplemental Figure 6 (neutrophils: CD45^+^CD11b^+^ly6G^+^, monocytes: CD45^+^CD11b^+^ly6G^–^, ly6C^hi^ monocytes: CD45^+^CD11b^+^ly6G^–^ly6C^hi^, and CD206^+^ macrophages: CD45^+^CD11b^+^ly6G^–^CD206^+^). Antibodies against CD45-BV421, CD11b-APC, Ly6G-APCcy7, Ly6C-AF700, and CD206-PE were used for flow cytometry (see Supplemental Table 2 for details). For cM cell separation, an immunomagnetic positive selection cell isolation kit was used (catalog 18970, EasySep).

### Macrophage isolation and culturing.

For BMDM culturing, BM was extruded from the femurs of euthanized mice and differentiated in culture media containing 50 ng/mL macrophage colony-stimulating factor (M-CSF) (PeproTech,) according to published procedures (41). Cells were used within 7–10 days of harvesting. PMs were isolated as previously described (42, 43).

### Measurement of mitochondrial function.

For Seahorse analysis, BMDMs were seeded overnight in quadruplicate at a density of 1 × 10^5^ cells per well on a pretreated XFe 24-well Seahorse microplate in RPMI 1640 medium containing l-glutamine, 10% FBS, and 50 ng/mL M-CSF and stimulated as specified. Prior to starting the OCR assay, cells were washed and incubated in XF Base Medium supplemented with 10 mM glucose, 1 mM sodium pyruvate, and 2 mM l-glutamine in a 37°C incubator without CO_2_ for 45 minutes. Oligomycin (ATPase inhibitor, 5 μM), FCCP (3 μM), and rotenone/antimycin A (1 μM /1 μM) were injected where indicated, and the OCR (pmoles O_2_/min) was measured in real time. Prior to starting the ECAR assay, cells were washed and incubated in XF Base Medium supplemented with 2 mM l-glutamine in a 37°C incubator without CO_2_ for 45 minutes. Oligomycin (5 μM), 2-deoxy-d-glucose (2-DG) (50 mM), and glucose (10 mM) were injected where indicated and the ECAR (mpH/min) was measured in real time. For flow cytometric analysis, MitoTracker Green (M7514, for total mitochondrial mass), tetramethylrhodamine methyl ester (TMRM) (I34361, for mitochondrial membrane potential), and MitoSOX (M36008, for mitochondrial ROS) staining were performed according to the manufacturer’s instructions (Invitrogen, Thermo Fisher Scientific). Data were acquired with an Aurora flow cytometer (Cytek) and analyzed with FlowJo software.

### Efferocytosis assay.

BMDMs were cultured in a 6-well plate and starved overnight (16 hours) before the assay. Aged RBCs from sheep’s blood (Colorado Serum Co., CS31113) were prepared by incubating the cells in PBS (~25% hematocrit) at 37°C for 4 days. After washing with PBS, RBCs were further labeled with CFDA-SE (CFSE) (Invitrogen, Thermo Fisher Scientific, V12883) for 30 minutes at 37°C. Subsequently, labeled RBCs were added to the macrophages and incubated for 15 minutes on ice followed by 3 hours at 37°C. After washing out the unbound RBCs and hypotonic lysis, cells were fixed and stained with CD206-PE antibody for flow cytometric analysis. In 1 experiment, BMDMs were treated with 2.5 μM or 0.5 μM cytochalasin B for 1 hour prior to addition of apoptotic RBCs for 8 hours. In separate experiments, mKO macrophages were treated with 1 μM Mito-TEMPO or vehicle for 30 minutes before addition of aged RBCs for 8 hours.

### Real-time PCR.

Total RNA from cells was extracted with TRIzol (Thermo Fisher Scientific) according to the manufacturer’s instructions. RNA was reverse transcribed into the first-strand cDNA using the Superscript First-Strand Synthesis Kit (Invitrogen, Thermo Fisher Scientific). cDNA transcripts were quantified using SYBR Green (Bio-Rad). mRNA levels were normalized to β-actin or 18s levels and are reported as the fold change over the control. The primer sequences are listed in Supplemental Table 3.

### Western blotting.

Electrophoresis of proteins was performed using the Tris-glycine SDS-PAGE nonreducing system according to the manufacturer’s protocol. Briefly, BMDMs were lysed in RIPA buffer (MilliporeSigma) with 1× protease and phosphatase inhibitors (MilliporeSigma) and stored at –20°C until analysis. Prior to electrophoresis, whole-cell lysates were diluted with 5× protein sample buffer (125 mM Tris-HCl [pH 6.8], 10% SDS, 50% glycerol, 0.06% bromophenol blue, and 1% β-mercaptoethanol) and incubated at 95°C for 5 minutes. Proteins were separated on 12% SDS-PAGE gels and transferred onto a PVDF membrane (MilliporeSigma). Primary antibodies against mouse NDUFS4 (3F11) and IL-1β (3A6) were from Thermo Fisher Scientific, and mouse β-actin (8H10D10) was from Cell Signaling Technology.

### Assessment of myofibroblast proliferation in vivo following MI.

Two doses of EdU (100 mg/kg) were i.p. injected at 24 hours and 9 hours, respectively, before tissue harvesting. Mice were then euthanized, and hearts fixed in 4% paraformaldehyde overnight. Tissues were then processed through a sucrose gradient (5%–30% sucrose), embedded in OCT, and prepared for 5 μm cryosectioning. For proliferation assays, Click-iT chemistry (Invitrogen, Thermo Fisher Scientific) was used to detect EdU positivity according to the manufacturer’s instructions.

### Tunel assay and immunofluorescence staining.

Heart tissues were harvested, embedded with OCT, and sectioned at 5 μm thickness. The Click-iT TUNEL Alexa Fluor 647 imaging assay (Thermo Fisher Scientific) was performed according to the manufacturer’s instructions and followed by staining with either anti-CD68 or anti-ly6G (conjugated Alexa Fluor 488) antibodies and Hoechst. Separate heart sections were stained with antibodies against fibronectin or α-SMA to detect fibroblast activation and with Hoechst to visualize the nucleus. Alexa Fluor 488– or Alexa Fluor 568–conjugated secondary antibodies directed against mouse or rabbit IgG were used to detect fibronectin or α-SMA fluorescence, respectively. Imaging analyses were performed using a Leica SPB confocal system. Details on the antibodies and fluorophores used are provided in Supplemental Table 4.

### Cytokine and chemokine determination.

Cytokine and chemokine levels were measured in plasma, heart homogenates, and culture supernatants obtained from BMDMs using ELISA kits according to the manufacturers’ instructions (R&D Systems and Thermo Fisher Scientific).

### Primary CF isolation.

CFs were freshly isolated as previously described (43, 44). Briefly, control hearts were perfused and digested by Langendorff perfusion with Krebs-Henseleit buffer (KHB) containing solubilized type II collagenase (2 mg/mL) and liberase blendzyme (0.4 mg/mL), and then incubated for 45 minutes at 37°C. For the macrophage and fibroblast crosstalk experiment, CFs were plated in DMEM with high glucose (Gibco, Thermo Fisher Scientific, 10566-016) and supplemented with 1% penicillin and streptomycin and 20% FBS, and then expanded to passage 3 for further analysis.

### Statistics.

Data are expressed as the mean ± SEM. The number of independent experiments is specified in the respective figure legends. Statistical analysis was performed with GraphPad Prism 8.0 (GraphPad Software) and SPSS 15.0 for Windows. Normal distribution of data was confirmed by a Shapiro-Wilk test. Statistical comparisons between 2 groups were conducted using an unpaired, 2-tailed Student’s *t* test. Differences in experiments with more than 1 independent variable were evaluated by 2-way ANOVA with post hoc Šidák’s or Tukey’s multiple-comparison test. Survival analysis was performed using the Kaplan-Meier method. Mortality was compared using the log-rank test. A *P* value of less than 0.05 was considered statistically significant.

### Study approval.

All animal procedures were performed in accordance with NIH guidelines and were approved by the IACUC of the University of Washington.

## Author contributions

MZ, SC, JD, GSO, and RT conceived and designed the study. SC, MZ, BZ, DB, OV, and AY performed experiments. SC and MZ analyzed data and drafted the manuscript. AS provided animals. All authors contributed intellectually to the project and approved the manuscript.

## Supplementary Material

Supplemental data

## Version 1 12/08/2022

In-Press Preview

## Version 2 02/15/2023

Electronic publication
