# Supplementary material for: Mitochondrial dysfunction in macrophages promotes inflammation and suppresses repair after myocardial infarction
Source: J Clin Invest. 2023 Feb 15;133(4):e159498. doi: 10.1172/JCI159498 (PMC9927948; doi:10.1172/JCI159498)
Supplement: Supplemental data [file jci-133-159498-s016.pdf]

Supplemental Figure 1. Myeloid-specific depletion of Ndufs4 impaired mitochondrial function and altered the responses to LPS in macrophages. (A) Ndufs4 expression level in peritoneal macrophages (PMs) from f/f and LysMcre$^{+/-}$, f/f (single cre), mice detected by western blot. **(B)** Oxygen consumption rate (OCR) was measured in PMs from LysMcre and mKO mice (n=4/group). **(C)** Analysis of mitochondrial mass using flow cytometry probes**.** BMDMs from WT and KO mice were stained with the mitochondrial probes MitoTracker Green. Representative MFI of mitochondrial mass content in macrophages (left). Quantification of MFI in WT and KO macrophages (right, n=4/group). Significant analyzed by student *t* test. **(D)** Mitochondrial membrane potential was measured by flow cytometry following incubation of BMDMs with TMRM. Flow cytometry histogram (left); Statistical analysis of mean fluorescence intensity (MFI) (right, n=4-5/group). **(E)** Scheme of glycolysis pathway and glycolytic enzymes. **(F-K)** Relative mRNA expression levels of genes involved in glucose uptake *Glut1* **(F)**, glycolysis, including *hk2* **(G)**, *pkm2* **(H)**, *aldoa* **(I)**, *gapdh* **(J)**, *ldha* **(K)** were detected by q-PCR in BMDM treated with LPS 10 ng/ml or vehicle for 6 h. n=3/group. **(M-S)** Gene expression involved in LPS induced signaling pathway in BMDM. mRNA levels of *tlr4* **(M)**, *cd14* **(N)**, *tlr9* **(O)**, *nlrp3* **(P)**, *ifnβ*1 **(Q)**, *cGas* **(R)** and *sting* **(S)** were detected by q-PCR (n=4/group). Data are presented as mean±SEM. Statistically significant difference analyzed by two-way ANOVA, $^*p < 0.05$, $^{**}p<0.01$, $^{***}p<0.001$. *Tlr4*=Toll-like receptor 4, *tlr9*=Toll-like receptor 9, *nlrp3*=NOD-, LRR- and pyrin domain-containing protein 3, *ifnβ*1= interferon beta 1, *cGas*= Cyclic GMP–AMP synthase, *sting*= stimulator of interferon genes.

Supplemental Figure 2. Echocardiographic measurements and scar size in MI/sham mice at day 30 post-surgery. (A) Male and **(B)** female mice heart rate were messered while taking

echocardiograph at day 30 post-mMI. n=3-10/group. Date are expressed as mean±SEM. Significant analyzed by two-way ANOVA. Male **(C)**, female LVID; d **(D)**. LVID; d= left ventricular internal dimension (diastole). Male **(E)** and female **(F)** LVID; s=left ventricular internal diameter in systole, n=5-10/group. **(G-H)** Scar size expressed as a percentage of the left ventricle in f/f, LysMcre and mKO mice at day 30 post-mMI. n=3-11/group male (**G**), female (**H**). Data are presented as mean±SEM. Statistically significant difference analyzed by two-way ANOVA, *p < 0.05, **p<0.01, ****p<0.0001.

Supplemental Figure 3. No difference of white blood cell counts among f/f, LysMcre and mKO mice. (A) Circulating neutrophils, **(B)** monocytes and **(C)** ly6C ^{hi} monocytes determined via flow cytometry in f/f, LysMcre and mKO mice. Cells was gated with $CD45^{+}CD11b^{+}ly6G^{-}$ for monocytes. Monocytes were further gated with ly6C. $CD45^{+}CD11b^{+}ly6G^{+}$ for neutrophils. n=5/ group. Data analyized by one-way ANOVA. Data analyized by one-way ANOVA.

Supplemental Figure. 4. Inhibition of macrophages effercytosis affects mRNA expression level in f/f BMDMs. BMDMs were treated with or without cytochalasin B (cyB) in either low dose of 0.5 nM, cyB (L) or high dose of 2.5 nM, cyB (H) before coculturing with apoptotic RBC or PBS for 8 h. mRNA level was detected by q-PCR. n=4/group. Data analyized by one-way ANOVA. **p<0.01, ***p<0.001, ****p<0.0001.

Supplemental Figure 5. Mt-TEMPO failed to normalize mitochondrial metabolism in mKO. **(A)** Representative tracings of oxygen consumption rate (OCR) of BMDMs from the indicated groups treated with mt-TEMPO 1μM or control (PBS) for 3 h. Vertical lines indicate time of addition of OA = oligomycin (5 μM), FCCP = carbonyl cyanide-p-trifluoromethoxyphenyl-hydrazon (3 μM), or R/A = rotenone/Antimycin A (1 μM/1 μM). **(B)**

Group average values at basal state and maximum respiration OCR. n=4/group. **(C)** Glycolysis measured by extracellular acidification rate (ECAR) in BMDMs treated with mt-TEMPO 1μM or control (PBS) for 3 h. **(D)** The average values of glycolysis and glycolytic capacity. n=3-5/group. Data analyzed by two-way ANOVA. *p<0.05, **p<0.01, ***p<0.001, ****p<0.0001.

Supplemental Figure 6. The gating strategies used for flow cytometry. (A) Gating strategy of MitoSOX staining related to Figure 1 E. **(B)** Gating strategy of CD80-FITC staining related to Figure 1 K and L. **(C)** Gating strategy related to Figure 3 A, F and Supplemental Figure 3A-C. **(D)** Gating strategy related to Figure 3 B, G, H and I.

Supplemental Table 1. Sample size for MI or sham surgery.

Supplemental Table 2. The antibodies used for flow cytometry list.

Supplemental Table 3. Sequences of primers used for q-PCR.

Supplemental Table 4. Antibodies and fluorophores used for immunofluorescence staining.

Supplemental Figure 1. Myeloid-specific deletion of Ndufs4 impaired mitochondrial function and altered the responses to LPS in macrophages.

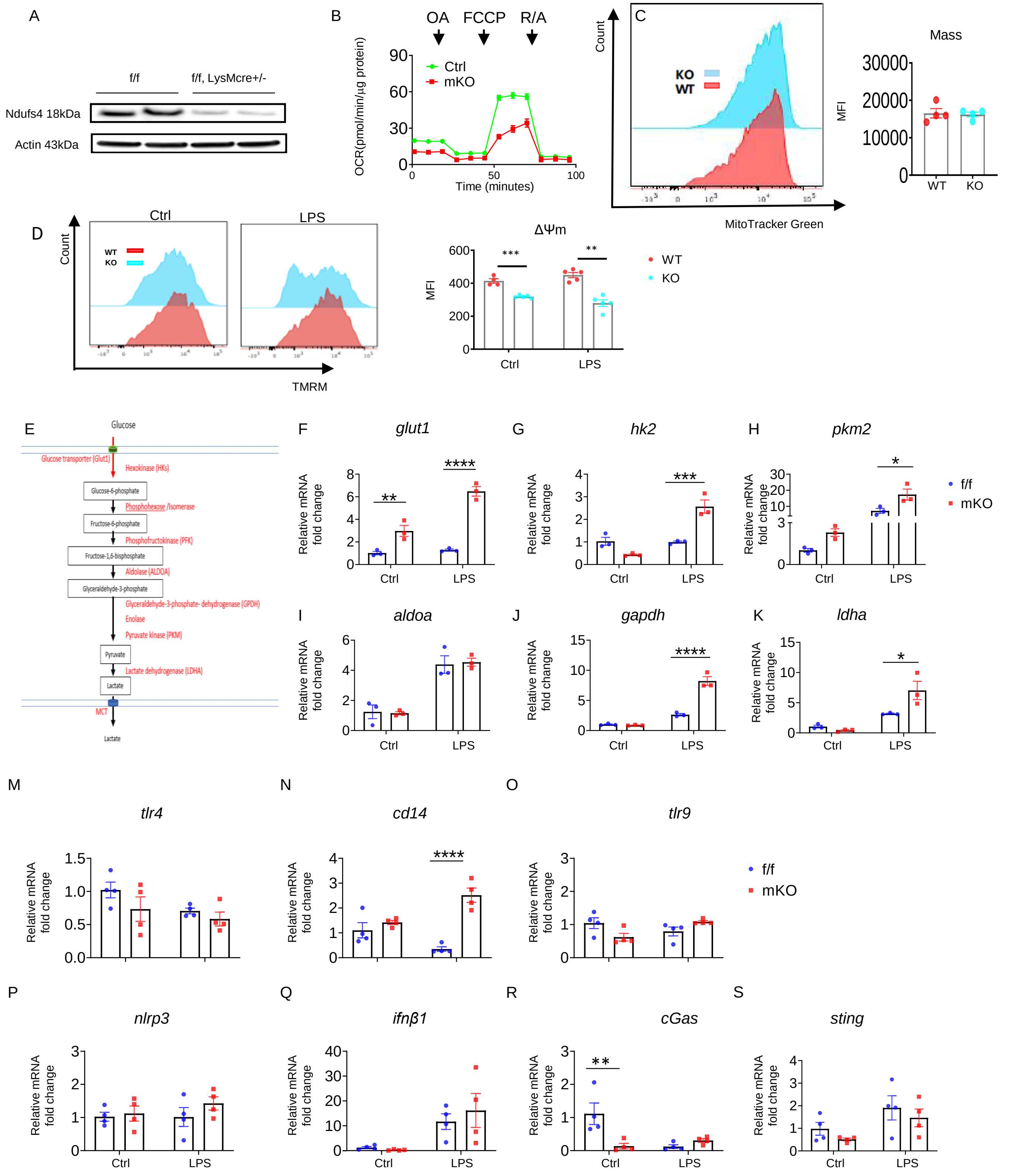

Supplemental Figure 2. Echocardiographic measurements and scar size in MI/sham mice at day 30 post surgery.

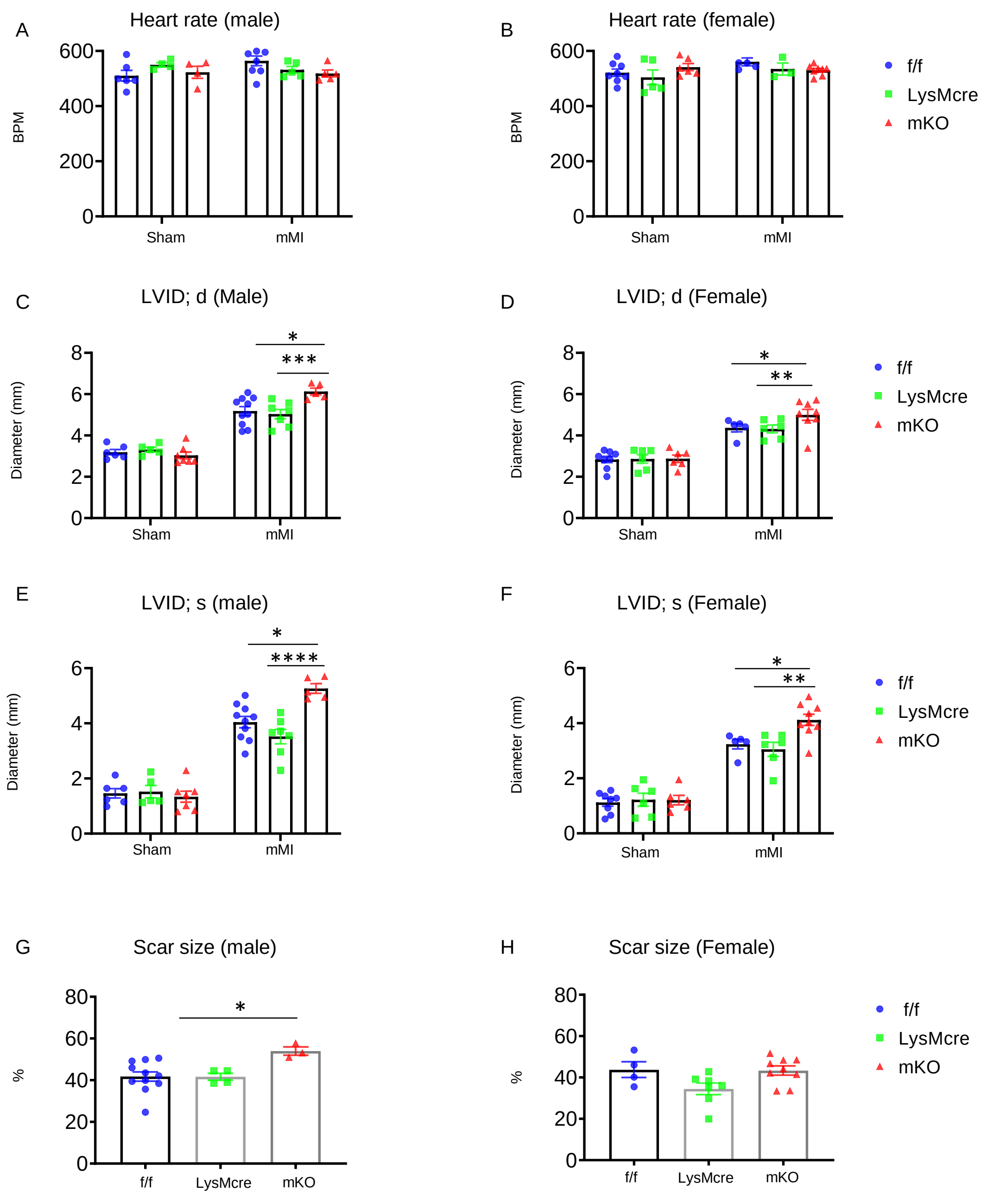

Supplemental Figure 3. No difference of white blood cell counts among f/f, LysMcre and mKO mice.

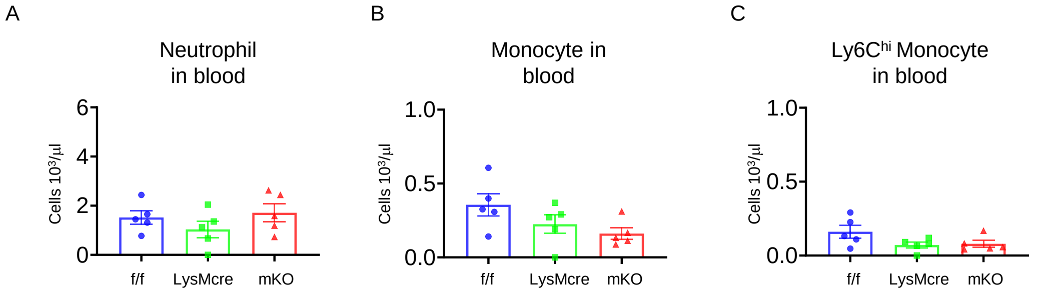

Supplemental Figure 4. Inhibition of macrophage efferocytosis affects mRNA expression level in f/f BMDMs.

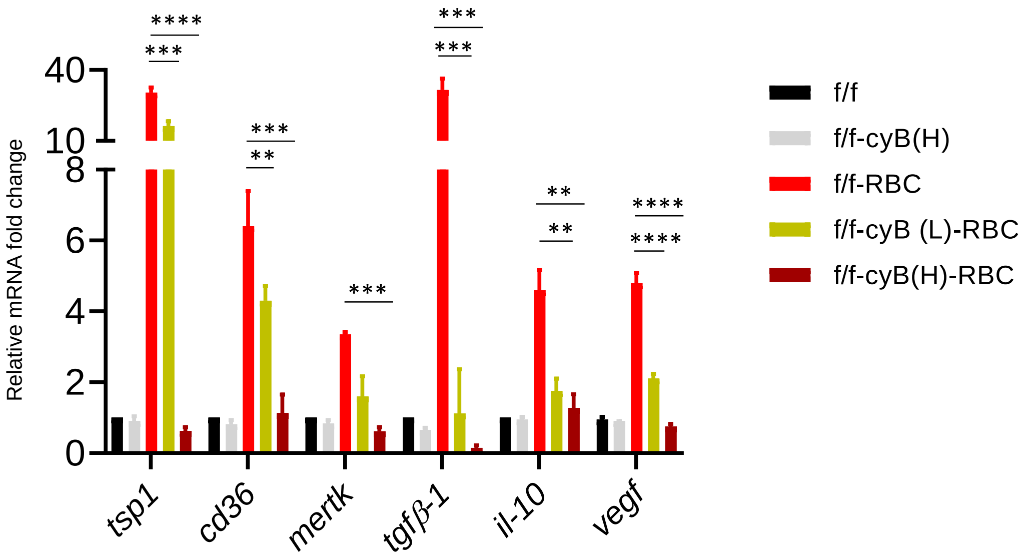

Supplemental Figure 5. mt-TEMPO failed to normalize mitochondrial metabolism of mKO.

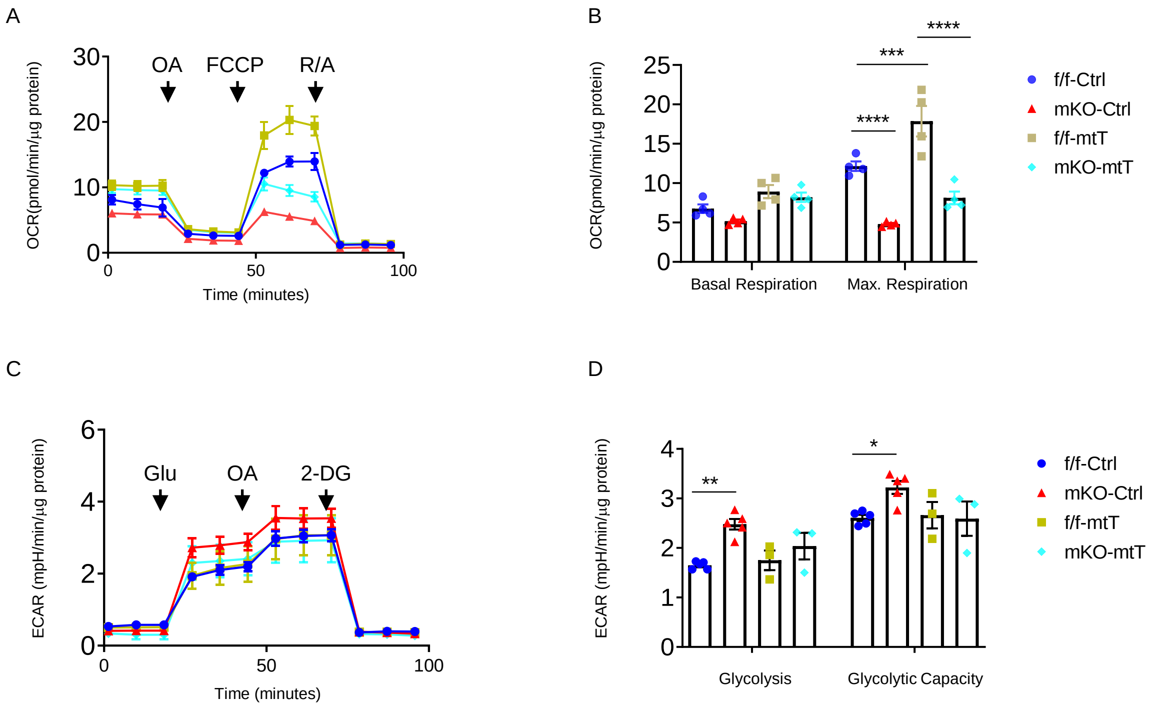

Supplemental Figure 6. The gating strategies used for flow cytometry.

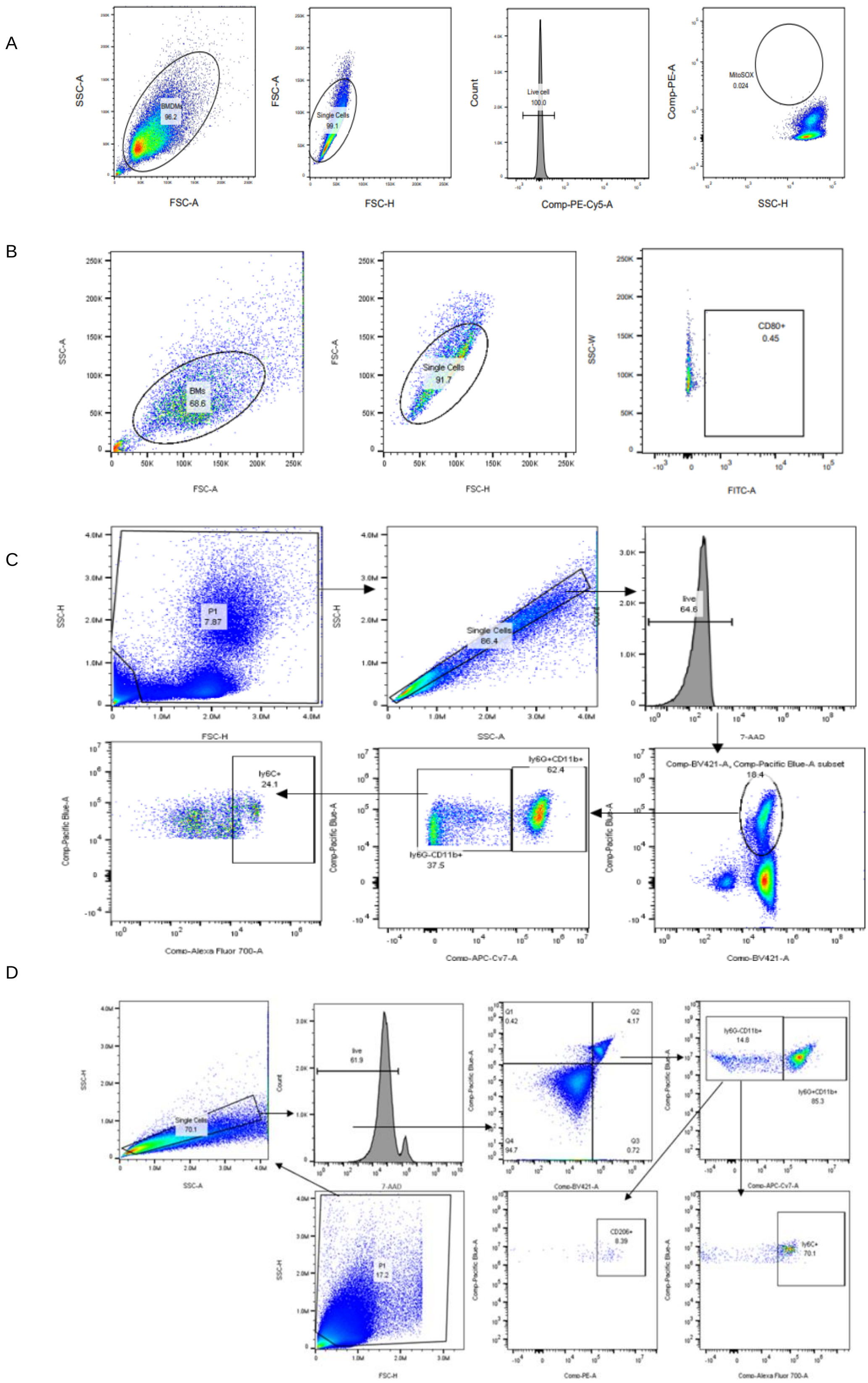

Supplemental Table 1. Sample size for MI or sham surgery.

	Male (MI)	Male (mMI)	Female (mMI)
f/f-Sham	6	6	8
LysMcre-Sham	7	7	6
mKO-Sham	7	7	6
f/f-MI/mMI	8	12	10
LysMcre-MI/mMI	13	9	8
mKO-MI/mMI	10	13	8

Supplemental Table 2. The antibodies used for flow cytometry list.

Dye and antibody for flow cytometry	Catalog no.	Company
Anti-CD45 Rat Monoclonal Antibody (Brilliant Violet® 421) [clone: 30-F11]	103133	BioLegend
APC/Cy7 anti-mouse Ly-6G [1A8]	127623	BioLegend
CD206 (MMR) Monoclonal Antibody (MR6F3), PE	12-2061-82	Thermo Fisher Scientific
CD80 (B7-1) Monoclonal Antibody (16-10A1), FITC	11-0801-82	Thermo Fisher Scientific
CD11b Pacific Blue anti-mouse/human [M1/70]	101211/101224	BioLegend
Anti-Ly-6C Rat Monoclonal Antibody (Alexa Fluor® 700) [clone: HK1.4]	128024	BioLegend
7-AAD Viability Staining Solution	420403	BioLegend
CD16/32 anti mouse	14-0161	invitrogen
MitoTracker Green	M7514	invitrogen
TMRM	I34361	invitrogen
MitoSOX	M36008	invitrogen

Supplemental Table 3. Sequences of primers used for real-time quantitative PCR (q-PCR).

Target gene	Primer sequence (Forward)	Primer sequence (Reverse)
18s	5’-CTT AGA GGG ACA AGT GGC G-3’	5’-ACG CTG AGC CAG TCA GTG TA-3’
ICAM1	5’-GTG ATG CTC AGG TAT CCA TCC A-3’	5’-CAC AGT TCT CAA AGC ACA GCG-3’
iNOS	5’-TTC ACC CAG TTG TGC ATC GAC CTA-3’	5’- TCC ATG GTC ACC TCC AAC ACA AGA-3’
CD36	5’- TCC TCT GAC ATT TGC AGG TCT ATC-3’	5’-AAA GGC ATT GGC TGG AAG AA-3’
MerTK	5’-GAG GAC TGC TTG GAT GAA CTG TA-3’	5’- AGG TGG GTC GAT CCA AGG-3’
TGF-β1	5’-TAC CAT GCC AAC TTC TGT CTG GG A-3’	5’-ATG TTG GAC AAC TGC TCC ACC TTG-3’
IL-10	5’-AAG GCA GTG GAG CAG GTG AA-3’	, 5’-CCA GCA GAC TCA ATA CAC AC-3’
TSP-1	5’- ACC GCA TTC CAG AGT CTG GC-3’	5’-ATG GGG ACG TCC AAC TCA GC-3’
VEGF	5’- GCA CAT AGA GAG AAT GAG CTT-3’	5’-CCC TCC GCT CTG AAC AAG GCT-3’
Glut1	5’-CAT CGT GGC CAT CTT TGG CTT TGT-3’	5’-GGA AGC ACA TGC CCA CAA TGA AGT-3’
HKs	5’-AGA TGT GGT CAC CTT GCT GAA GGA-3’	5’-GCC AAC AAT GAG GCC AAC TTC ACA-3’
PFK	5’-GGT CCG AGT TGG TAT CTT CAC-3’	5’-ACT TCC AAT CAC TGT GCC AC-3’
ALDOA	5’-CCC CAA GTT ATC AAG TCC AAG G-3’	5’-GTT CAG ACA GCC CAT CCA G-3’
GAPDH	5’-CTT TGT CAA GCT CAT TTC CTG G-3’	5’-TCT TGC TCA GTG TCC TTG C-3’
PKM	5’-CTG AAG GCA GTG ATG TGG CC3’	5’-ACC CGG AGG TCC ACG TCC TC-3’
LDHA	5’-GCT CCC CAG AAC AAG ATT ACA G-3’	5’-TCG CCC TTG AGT TTG TCT TC-3’
TLR4	5’-ACC TGG CTG GTT TAC ACG TC-3’	5’-CTG CCA GAG ACA TTG CAG AA-3’
TLR9	5’-ACT GAG CAC CCC TGC TTC TA-3’	5’-AGA TTA GTC AGC GGC AGG AA-3’
CD14	5’-AGC ACA CTC GCT CAA CTT TTC-3’	5’-GCC CAA TTC AGG ATT GTC AGA C-3’
NLRP3	5’-AGA AGA GAC CAC GGC AGA AG-3’	5’-CCT TGG ACC AGG TTC AGT GT-3’
TNFR1	5’-CCG GGC CAC CTG GTC CG-3’	5’-CAA GTA GGT TCC TTT GTG-3’
TNFR2	5’- GTC GCG CTG GTC TTC GAA CTG-3’	5’-GGT ATA CAT GCT TGC CTC ACA GTC-3’
Caspase8	5’-CTC CGA AAA ATG AAG GAC AGA-3’	5’-CGT GGG ATA GGA TAC AGC AGA-3’
Collagen Ia	5’- GCT CCT CTT AGG GGC CAC T -3’	5’- CCA CGT CTC ACC ATT GGG G-3’
α-SMA	5’-GAC GTA CAA CTG GTA TTG TG-3’	5’-TCA GGA TCT TCA TGA GGT AG-3’
IFNβ1	5’-CCC TAT GGA GAT GAC GGA GA-3’	5’-CTG TCT GCT GGT GGA GTT CA-3’

Supplemental Table 4. Antibodies and fluorophores used for immunofluorescence staining.

Antibody clones and fluorophores	Catalog no.	Company
Click-it Tunel Alexa Fluor 647	C10618	Thermo Fisher Scientific
Alexa Fluor® 488 anti-mouse CD68 Antibody	137011	Biolegend
Alexa Fluor® 488 anti-mouse Ly-6G Antibody	127625	Biolegend
Fibronectin Polyclonal antibody	15613-1-AP	Proteintech
Anti-Actin, α-Smooth Muscle antibody, Mouse monoclonal	A5228	Sigma
Hoechst 33342	H3570	Invitrogen
Goat Anti-Mouse IgG H&L (DyLight® 488)	ab96879	abcam
Goat anti-Rabbit IgG (H+L) Cross-Adsorbed Secondary Antibody, Alexa Fluor™ 568	A11036	Invitrogen
